# Meditation: A Double-Edged Sword—A Case Report of Psychosis Associated with Excessive Unguided Meditation

**DOI:** 10.1155/2022/2661824

**Published:** 2022-10-22

**Authors:** Sripathi Santhosh Goud

**Affiliations:** Indlas VIMHANS Hospital, India

## Abstract

Meditation is gaining a lot of popularity nowadays because of the associated benefits on both mental and physical health. There is ample literature available on the benefits of meditation. However, there are cases of psychosis reported sporadically in individuals who indulge in excessive unguided meditation. Herein, we report a patient who presented with schizophrenia after doing excessive meditation.

## 1. Introduction

The practice of meditation is known to mankind since ages. Yet, it is hard to define meditation because of a wide range of dissimilar methods practiced in different cultures in the name of meditation. Despite these different practices, the ultimate objective of meditation is mental well-being and psychophysical relaxation. Walsh and Shapiro thus defined meditation as a family of self-regulation practices that focus on training attention and awareness in order to bring mental processes under greater voluntary control and thereby foster general mental well-being and development and specific capacities such as calm, clarity, and concentration [1]. Meditation practices can be divided into two broad categories: concentrative meditation and mindfulness meditation. Concentrative meditation also known as focused-attention meditation includes paying attention to breathe, a mantra, an idea, or a feeling, i.e., focusing attention on a single point. Mindfulness meditation is an open method that involves developing a present-centered and unattached mode of observation toward all sensory phenomena, including thoughts. Transcendental meditation is a kind of concentrative meditation, involves using of a mantra, and is practiced for 20 minutes twice per day while sitting comfortably with one's eyes closed.

Meditation has been studied as a useful aid in the treatment of conditions like chronic pain, hypertension, anxiety disorders, and insomnia. Mindfulness meditation is found to be effective in the treatment of depression and drug addiction [2]. The effects of meditation come through increase in blood flow to the brain regions particularly prefrontal cortex(PFC), cingulated gurus, and hypothalamus along with alterations in various neurochemicals like dopamine(DA), serotonin, glutamate, gamma-aminobutyric acid(GABA), dimethyltryptamine (DMT), N-acetylaspartylglutamic acid (NAAG), and corticotropin-releasing hormone(CRH) [3]. Sometimes, this alteration results in dysregulation of neurochemicals resulting in psychosis like states. Previous case reports suggested that those practicing concentrative meditation are more prone for developing psychosis than those practicing mindfulness meditation. Here, we report a case of a 40-year-old female who presented with schizophrenia like psychotic illness after excessive indulgence in concentrative meditation.

## 2. Case Report

A 40-year-old female patient was involuntarily brought by the husband and mother with chief complaints of meditating for 14-18 hours a day, neglecting food, sleep, and self-care. She had stopped doing household chores and instead used to sit for hours together with her eyes closed while mumbling to self, since last 4 years. The patient was interviewed in an empathetic and nonjudgmental way to make her comfortable and share her story. She was introduced to a meditation guru who teaches transcendental meditation by one of her family friends. After that, the patient never met the guru. Though the guru instructed her to practice meditation in her free time for a limited duration (20 minutes twice per day), she gradually increased time spent on meditation from initial one hour to 14-18 hours. Later, she would watch videos related to meditation on television and Internet for the rest of her day. When asked, she told that she felt very much relaxed and felt a sense of detachment from her body and a great sense of pleasure when she meditated. Hence, she increased her time spent on meditation. When enquired about her mumbling behaviour, she told that she was talking to her guru whose voice she could only hear. The voice of her guru ordered her to practice the meditation throughout the day to activate inner chakras and get special powers. She would sit in her room and talk to the guru through “telepathy” according to the patient. She also reported that she got the power to see the future of others with her third eye, and she reported that meditation gave her these superpowers. On inquiry, she told that she can travel through time and space and can communicate with the aliens while meditating. She started speaking in gibberish manner saying that she was replying to aliens in their language. She also told that few aliens were following her in the form of humans from the last few years. On further probing, she stated that those were bad aliens who came to kidnap and harm her.

When questioned about her reduced sleep, food intake, and her poor self-care, she told that meditation itself keeps her healthy, fit, and clean. She was refusing to have sex with her husband and was demanding for divorce. When questioned about this, she answered that she had a special bonding with her guru and she considers her guru as her “soul mate.” She could perceive “the signals” from her guru who stays away from her, as her soul meets with the soul of her guru in universal space and she equated this to sex between their souls. She was physically aggressive towards her family members if they asked her to stop meditating. She stopped attending any social gatherings saying people will steal her powers if she goes out of the house. The course of the illness was insidious in onset and continuous and progressive in nature. The patient was reluctant to consult any mental health professional, so she was brought forcefully.

She had no past history of significant psychological or medical illness. Her mother and maternal uncle were diagnosed with unspecified psychoses and received treatment in the past. Premorbidly, the patient was well adjusted. She was a graduate and used to work as a primary school teacher till 4 years back. She got married at the age of 25 years and has 2 kids. She used to take care of the family and kids before the onset of the illness. Her husband works as a government employee, and their relationship was cordial before the onset of the illness as per the husband.

On examination, the patient was malnourished and unkempt. She was continuously muttering to herself throughout the interview. Her speech was disorganized sometimes. She reported perceptual experiences like auditory hallucinations, telepathy, and clairvoyance. In thought content persecution, grandiosity and magical thinking were found. Her test and personal judgment were impaired. She had no insight into her illness. Provisionally, she was diagnosed with schizophrenia as per Diagnostic and Statistical Manual of Mental Disorders 5^th^ edition (DSM5). She scored 78 on Brief Psychiatric Rating Scale (BPRS) and 6 (severely ill) on the Clinical Global Impression-Severity (CGI-S) Scale. Her responses on the Rorschach inkblot test were suggestive of schizophrenia. Her blood investigations were unremarkable except for low hemoglobin. She refused using any psychotropic substances, and her urine drug screen was negative for any drugs. Computerized tomography (CT) of the brain revealed no abnormality.

She was admitted to the hospital and started on tablets olanzapine, lorazepam, and iron supplementation. As the patient was very much symptomatic, she refused to take medication and food. Six sessions of modified electroconvulsive therapy (M-ECT) were given. The patient showed improvement and started to accept food and tablets. She was discharged after 20 days from the hospital. The patient was stabilized on 15 mg of oral olanzapine. Her BPRS score came down to 32 and CGI-S score to 3 at the time of discharge. She was advised to refrain from any kind of unguided meditation. Family members of the patient were doubtful about the compliance with treatment at home. She was shifted to olanzapine depot injection and currently receiving 405 mg of olanzapine palmitate on monthly basis and maintaining well with regular follow-ups.

On Naranjo's scale, the patient had scored 5 indicating “probable role” of meditation in causing psychosis.

## 3. Discussion

Individual case reports of psychosis associated with meditation were reported mostly from western countries. In India, Sethi and Bhargava reported 2 cases of psychosis associated with meditation. One of the two patients was a known case of psychosis, and each psychotic episode started after attending a religious retreat. The second patient had no past or family history of psychotic illness. The authors concluded that along with meditation, sleep deprivation also resulted in psychotic breakdown [4]. Charan et al. in their recent case series had analyzed the possible risk factors associated with meditation and psychosis. According to them, family history of psychiatric illness, individuals with cluster A personality traits, type of the meditation practiced, and duration of meditation were found to be related to the development of psychosis in vulnerable individuals [5]. A variety of complex neurochemical changes like increased serotonin (5-HT2) receptor activation and abnormally elevated DA, DMT, and NAAG levels were seen in individuals developing psychosis after doing meditation. DMT and NAAG were having hallucinogenic effects, whereas abnormally elevated DA in temporal lobes was linked to formation of delusions [3].

In the present case, the patient had family history of psychosis in mother and maternal uncle. She was premorbidly well adjusted and functional. After starting meditation, the patient spent most of her time in a single room, away from her friends and family members, which resulted in sensory deprivation. Reduced food intake resulted in a state of malnutrition. Along with poor nutrition, sleep deprivation might have also contributed for development of her psychotic illness. Because of the lack of insight, the patient refused to seek help that resulted in further deterioration of her clinical condition. Finally, when she was insisting for divorce, the family members brought her to the hospital involuntarily.

Antipsychotics are the main stay of treatment for meditation-induced psychosis. Both first- and second-generation antipsychotics are found to be equally effective. The patients should be discouraged from practicing unsupervised meditation to prevent further exacerbation of psychosis.

## 4. Conclusion

Meditation is a double-edged sword. When properly done under the guidance of a trainer, it helps to improve both mental and physical health. Though cause effect relationship cannot be established with the available research data, meditation was found to be associated with development and/or exacerbation of psychotic disorders in predisposed individuals. Future research should consider longitudinal follow-up studies to further explore a potentially causal role of meditation in the development of psychosis. When therapists decide to offer a meditation-based intervention for their clients, careful weighing of the potential risks and benefits of that intervention should be done.

## Data Availability

No data were used to support this study.
